# Patient-reported outcome measures (PROMs) use in post-stroke patient care and clinical practice: a realist synthesis protocol

**DOI:** 10.1186/s13643-021-01682-w

**Published:** 2021-04-28

**Authors:** A. Smith, J. Hewitt, T. J. Quinn, M. Robling

**Affiliations:** 1 Division of Population Medicine, Cardiff University, Cardiff, UK; 2 Institute of Cardiovascular and Medical Sciences, University of Glasgow, Glasgow, UK; 3 Centre for Trials Research, Cardiff University, Cardiff, UK

**Keywords:** Stroke, Systematic review, Patient-reported outcome measure, PROM, Realist synthesis, Patient care, Feedback

## Abstract

**Background:**

There is growing interest in the use of routine patient-reported outcome measures (PROMs) to influence the care of individual patients with stroke. However, there are significant gaps in our understanding as to how PROMs influence post-stroke patient care and clinical practice. This is due to factors including the number of purported uses for PROMs and that PROMs are complex interventions, which attempt to stimulate varied actions or behaviours. Therefore, the objective of this realist synthesis is to offer theory-based explanations as to how PROMs influence post-stroke clinical practice and patient care.

**Methods:**

This is a protocol for a realist synthesis, which involves three distinct phases: theory building (phase 1), theory testing and refinement (phase 2) and synthesis (phase 3). Phase 1 will develop initial rough programme theories (IRPTs), through literature searches (from January 2000 onwards) of MEDLINE, EMBASE, PsycINFO, CINAHL, Cochrane Library and the grey literature. Only secondary sources will be included that contribute to the development of IRPTs. Only two IRPTs, prioritised by the stakeholder group, will be taken forward to be tested and refined during phase 2. Further novel searches will be employed in phase 2, utilising the same criteria as phase 1; however, phase 2 searches will not utilise grey literature searches, and only primary research studies that contribute to the refinement of programme theories under investigation will be included. Two independent reviewers will screen and select all returned results. The reviewers will code and annotate relevant sources, resulting in ‘fragments’ to be extracted and graded based on the richness of their contribution to explanation and causal insight. Further, these fragments will be organised into ‘Context-Mechanism-Outcome’ configurations. Phase 3 of the review will involve the synthesis of context-mechanism-outcome configurations to form middle-range theory-based explanations and developed logic models for stakeholders to understand how PROMs in post-stroke clinical practice and patient care work for whom, how and under what circumstances.

**Discussion:**

The resulting realist synthesis will provide guidance on the implementation of PROMs within routine post-stroke clinical practice and patient care and act as a touchstone for further testing and refinement of PROMs programmes.

**Systematic review registration:**

PROSPERO CRD42020138649.

**Supplementary Information:**

The online version contains supplementary material available at 10.1186/s13643-021-01682-w.

## Background

The impact of a stroke or the outcome of treatment is most commonly determined from the perspectives of healthcare professionals or the healthcare system but seldom from the perspectives of stroke survivors. In contrast to individual patient-level clinician-administered assessments, healthcare systems gather data relating to the adherence to evidence-based key performance indicators (KPI), such as those advocated in the UK by the Royal College of Physician’s National Clinical Guidelines for Stroke [1], which are important indicators of care quality and are correlated with improved outcomes [2]. In spite of initiatives to routinely collect data from the perspective of stroke survivors, such as the recent inclusion of a patient-reported outcome measure (PROM) within the Australian Stroke Clinical Registry (AuSCR) [3], what is often missing in the routine post-stroke outcome landscape is the patient perspective.

Patient-reported outcomes (PROs) and patient-reported outcome measures (PROMs) offer the opportunity for patients to convey their perception of health status, quality of life or the impact of treatment on their health or quality of life [4]. It is necessary to draw a distinction between PROs and PROMs, in that PROs refer generally to outcomes that are reported by the patient, whereas PROMs are standardised, psychometrically valid and reliable tools [5]. In light of the opportunities afforded by PROMs, there is a growing interest in the utility of PROMs, within stroke clinical practice and patient care [6], which is the focus of this review.

The literature pertaining to PROMs and stroke can be grouped under two major areas of research, firstly where PROMs are a study outcome [7] and secondly as studies relating to the development or evaluation of a PROM including its psychometric properties [8]. Outside of these two major areas, there are a further number of relatively smaller, but emerging areas of research. One such growing area of interest is those studies investigating PROM utility and its impact within routine clinical practice and patient care. In line with previous research [9, 10], this review will regard PROMs within routine clinical practice and patient care as a complex intervention. The defining characteristic of PROMs as a complex intervention is how PROMs are used flexibly to facilitate or stimulate actions or behaviours to guide patient care based on the data collected by the PROMs. Responding to the conception of PROMs used as a complex intervention, this review will focus on the actions that are stimulated by PROMs feedback within routine stroke clinical practice and patient care.

The literature, relating to the utility of PROMs within clinical practice and patient care, from differing health conditions, has identified many diverse uses for PROMs [10]. This realist synthesis is focused to answer the review question ‘how do Patient-Reported Outcome Measures (PROMs) influence post-stroke clinical practice and patient care?’ Therefore, with a multitude of potential uses for PROMs, understanding how PROMs might positively influence post-stroke clinical practice and patient care is both timely and necessary. Thus, this paper presents a protocol for a realist synthesis, which is a review methodology specifically designed, to interpret and synthesise the available evidence from complex interventions [11], such as PROMs, into refined theory-based explanations regarding their use. Therefore, this review will act as a touchstone for the clinical and research community to enact refinements and shifts in the emphasis of ongoing and novel PROMs programmes within post-stroke patient care and clinical practice.

## Methods

### Aims and objectives

This protocol is being reported in accordance with the reporting guidance provided in the Preferred Reporting Items for Systematic Reviews and Meta-Analyses Protocols (PRISMA-P) statement [12] (see checklist in Additional file 1). This protocol has been registered within the International Prospective Register of Systematic Reviews (PROSPERO) database (registration ID: CRD42020138649). The final review will be reported in accordance to the Realist And Meta-narrative Evidence Syntheses: Evolving Standards (RAMESES) guideline [13].

### Phase 1—theory building

The first aim is to build initial rough programme theories (IRPTs) [14], which are a set of preliminary broadly conceived theories, relating to the shared assumptions underpinning the use of PROMs within post-stroke clinical practice and patient care. This aim will be met by the following objectives:

I. To comprehensively search and identify all relevant papers at the level of secondary literature such as reviews, editorials, comments, and letters. This will include established research databases and the grey literature.
II. Identify and catalogue the various proposed and current uses of PROMs within the context of stroke clinical practice and patient care.
III. To outline, in the form of initial rough logic models (IRLMs), the current and proposed uses of PROMs at the level of stroke clinical practice and patient care. The models will detail decision-making sequences ranging from the micro (individual) to meso (group) levels and will include the full PROMs process from their collection, interpretation and the clinical actions arising from PROM data.
IV. To develop a set of initial rough programme theories (IRPTs) [14] from the identified literature, supported by the IRLMs, relating to the different programme theories of PROMs used at the level of stroke clinical practice and patient care.
V. To convene a stakeholder group consisting of stroke survivors and health professionals involved in post-stroke care, to prioritise two IRPTs, from the IRPTs identified in phase one, for the purpose of testing and refinement in phase two.

### Phase 2—theory testing and refinement

Building on the aim and objectives of the theory-building aspect of this realist synthesis, the aim of the second phase is to test and refine two newly developed IRPTs from phase one, prioritised by the stakeholder group, using primary research studies. The aim of phase two, theory testing and refinement, will be achieved by the following objectives:

I. To comprehensively search and identify all relevant papers, using a novel search strategy, from that employed in phase one, based on the prioritised IRPTs, at the level of primary research studies. Additionally, to catalogue and model, any novel PROMs uses that arise from the evidence identified in the theory testing searches.
II. To test and refine the prioritised IRPTs via the primary research studies identified in the theory-testing searches.
III. To refine the IRLMs of the previously prioritised IRPTs, to create refined logic models, using the literature identified via the theory-testing search strategies
IV. To identify the mechanisms and contexts through which the identified programme theories operate that either promotes or constrains successful use of PROMs at the level of stroke clinical practice and patient care. This will include both successful and unsuccessful PROM utilisation including the intended and unintended outcomes of the programme theories identified.

### Phase 3—synthesis

The final aim of this realist synthesis is to synthesise the evidence into programme theories, thereby, refining our understanding of how PROMs work, in what contexts and for who. Thus, assembling and interpreting the evidence for the purpose of producing theory-based explanations of PROMs use in post-stroke clinical practice and patient care. Specific objectives are as follows:

I. To adjudicate and synthesise competing claims, contained within the identified evidence, for the purpose of constructing theory or theories with the greatest explanatory power.
II. To offer theory-based explanations, utilising inference to the best explanation [15] approach, as to which programme theories relating to the collection, interpretation and clinical actions arising from PROMs data best explain the outcome patterns across the available literature.
III. To develop logic models based on the programme theories of PROMs use, tested and refined in phase 2, to represent diagrammatically the procedures and decisions involved in the use of PROMs in post-stroke clinical practice and patient care

## Review methodology

### Justification of the realist approach

The hallmark question of realist synthesis is ‘What works for whom in what circumstances?’ [8], which encapsulates its inquiry methodology beyond the purported efficacy of an intervention toward building theory-based explanations. This is an apparent advantage when analysing complex interventions such as PROMs, which are many faceted interventions and whose efficacy does not always simply transfer across multiple contexts. To achieve this, realist synthesis seeks to open the ‘black box’ of an intervention, drilling down into the underlying mechanism or mechanisms, which produce the positive or negative outcome patterns or in realist terms ‘demi-regularities’ [11] as observed in the literature. Thus, complex interventions such as PROMs can attempt to draw on multiple mechanisms to stimulate actions or behaviours based on PROM data. However, mechanisms operate in a pre-existing context, which is best thought of as plural ranging from the micro (individual), meso (groups) to the macro (institutions) [16, 17]. In the case of PROMs use, context can be seen to run from the bedside, through multidisciplinary team feedback and, as aggregated scores, to the hospital and wider healthcare system. The most important aspect of the context-mechanism relationship is that certain mechanisms will enable certain outcomes in specific contexts and constrain the same outcomes in other contexts. Hence, for Pawson and Tilley [11], it is the relationships between the context, mechanism and outcome (CMOs) that gives rise to theory-based explanations about the functioning of an intervention.

Realist synthesis is a mixed-methods review methodology, thereby allowing for the simultaneous review and synthesis of quantitative and qualitative research. This mixed-methods approach becomes a necessity when applied to the methodologically heterogeneous evidence base of complex interventions such as PROMs, by allowing for the investigation of the many facets of the intervention as they have been investigated by differing research methodologies. Consequently, realist synthesis is a strong fit for the area of this review and is well aligned with the review question to provide explanations as to why certain programme theories related to the use of PROMs post-stroke work in these respects, for these subjects, in these kinds of situations [11].

### Phase 1: theory building

The initial phase within this realist synthesis is organised around meeting the aim of theory building by firstly identifying and cataloguing the various proposed and actualised uses of PROMs within the context of post-stroke clinical practice and patient care. In line with previous realist syntheses, the first phase of theory building has been completed in conjunction with the development of the protocol. The identification and cataloguing contributed to the building of initial rough logic models (IRLMs) detailing the sequences necessary to the intervention to produce the outcomes intended or otherwise. The models subsequently facilitate the development of what Shearn et al. [14] have termed initial rough programme theories (IRPTs) that will guide the subsequent phases of this realist synthesis.

#### Literature search: theory building

The theory-building searches consisted of multicomponent searches, see Table 2, organised under three major concepts: A ‘Stroke’, B ‘PROMs’, C ‘Evidence Type’. In relation to concept A ‘stroke’, a validated search syntax was used to enhance search rigour and consistency. In contrast, the search strategy for concepts B and C were refined through an iterative process to identify further terms and synonyms to those outlined in Table 1, which was developed for OVID Medline. The culmination of this process was the combination of the concepts with the ‘AND’ operator, to retrieve secondary evidence that relates to PROMs use post-stroke. Secondary evidence here refers to evidence which is not developed through a primary research study, i.e., reviews, letter, comments, and opinions pieces. The search syntax was translated to meet the requirements of the following databases, MEDLINE, EMBASE, PsycINFO (OVID) CINAHL (Ebsco) and Cochrane. The theory-building search also included searches of the grey literature such as national clinical guidelines and the open access areas of websites related to professional bodies (Additional file 2.) of professions routinely involved in the care or treatment of stroke survivors. The searches of the grey literature were conducted utilising the ‘advanced search’ function of Google Search and limited to specific domains for searches of websites of professional bodies. In line with RAMASES publication standards for realist syntheses [15], each part of the searching process was documented accurately to ensure transparency, and the final search syntax will be published alongside the result paper.

**Table 1:** Indicative search terms—phase 1: theory-building searches

	A—Stroke	B—PROMs	C—Evidence type
Index terms	cerebrovascular disorders/ basal ganglia cerebrovascular disease/ brain ischemia/ carotid artery diseases/ cerebral small vessel diseases/ intracranial arterial diseases/ “intracranial embolism and thrombosis”/ intracranial hemorrhages/ stroke/ brain infarction/ stroke, lacunar/ vasospasm, intracranial/ vertebral artery dissection/	Patient Reported Outcome Measures/ Self Report/ Quality of Life/ Health Status/ Patient Satisfaction/	Comment/ Letter/ Editorial/ News/ Newspaper Article/ “Review”/ “Systematic Review”/ Practice Guidelines as Topic/
Text word terms	brain$ cerebr$ cerebell$ intracerebral intracran$ parenchymal intraparenchymal intraventricular infratentorial supratentorial basal gangli$ putaminal putamen posterior fossa hemispher$ subarachnoid **adj5** h?emorrhag$ h?ematoma$ bleed$	Patient$ Report$ **adj3** Outcome$ Measure$ Patient Reported Outcome Measure$ Patient Reported Outcome$ Patient report$ **adj3** outcome$ PROM? QoL or HRQoL or HQoL or quality of life adj assess$ or questionnaire$ or screen$ or index or indices or instrument$ or inventor$ or measure$ or scale$ or tool$	

#### Inclusion and exclusion criteria: theory-building literature search

The inclusion and exclusion criteria outlined in this section apply solely to the theory-building searches, which are detailed in Table 2. For the purpose of conceptual clarity, the term ‘post-stroke’ refers to the whole stroke pathway from acute admission to post-discharge community-based follow-up. The inclusion of the whole stroke pathway is due to the limited and heterogeneous studies investigating PROM utility within routine clinical practice and patient care as identified in scoping searches. Moreover, there is a lack of definitive guidance on the optimum timing of post-stroke PROM collection. In light of the multi-disciplinary nature of post-stroke care and treatment, the terms ‘clinical practice’ and ‘patient care’ refer to the clinical practice of all healthcare professionals who are involved in post-stroke care and treatment.

**Table 2:** Inclusion/exclusion criteria—phase 1: theory-building searches

Inclusion	Exclusion
Evidence relates to a population clinically diagnosed with any or a mixture of the following conditions: cerebral infarction, non-traumatic intracerebral haemorrhage.	Evidence relates to a study population solely focused on any of the following conditions: transient ischaemic attack (TIA), traumatic brain injury, hypoxic brain injury, subarachnoid haemorrhage.
Evidence relates to an adult stroke population at any period post-clinical diagnosis.	Evidence relates to a paediatric stroke population.
Evidence relates to studies focused on the use of patient-reported outcome measures within routine clinical practice or care at the micro- and/or meso-levels. (incl. pragmatic studies)	Evidence relates to studies concentrating on the development of a PROM.
Evidence relates to PROMs use at the level of the individual.	Evidence relates to studies concentrating on other patient-reported tools such as patient-reported experience measures (PREMs), non-standardised patient-reported outcomes or tools with only a partial patient-reported element.
Evidence must relate to studies where PROM score interpretation and/or feedback is necessary.	Evidence relates to studies concentrating on secondary testing or comparative testing of PROMs.
English language or high-quality translation available.	Evidence relates to studies concentrating on the psychometric properties of a PROM or PROMs (e.g. validity and reliability).
Any secondary literature, e.g. literature reviews, comments, editorials.	Evidence relates to studies involving the aggregation of individual PROM scores.
	Evidence relates to studies in which PROMs are an outcome of a research study, including the evaluation of an intervention or observational research exploring trends in quality of life.
	Any primary research studies understood as involving primary data capture to answer a novel hypothesis either via experimental or observational design (*this type of study design is reserved for phase 2: theory testing*)
	Date of publication pre 01/01/00 due to the significant changes to stroke care and services.

Beyond the scope of this review, and included in the exclusion criteria, are the uses of PROMs outside of routine clinical practice or patient care settings. Moreover, also excluded are studies of the aggregate uses of PROMs, which involve the analysis of aggregated PROMs scores. Thus, the review will operate across the micro-level (the individual actors, such as healthcare professionals and stroke survivors and the decisions they make) and meso-level (group actions and decisions such as those undertaken by the multidisciplinary healthcare team) [8, 9], where the care or treatment of an individual is the primary focus. Literature prior to 2000 will be excluded due to the number of changes in stroke care in the two decades subsequent with the introduction of thrombolytic treatments and the resulting reorganisations of stroke care. Whilst specific study methodologies were not excluded per se, primary research studies were excluded from this initial, theory-building literature search. The privileging of secondary sources of evidence allows for primary research studies to be reserved for the theory-testing searches and avoids any circularity of reasoning, as primary research studies contribute empirical evidence to directly test and refine the programme theories, which is the aim of phase two of this review.

#### Data extraction

Data extraction will involve the identification and coding of actual and proposed uses of PROMs within papers found to meet the inclusion criteria. The coded ‘PROMs uses’ will be extracted to an Excel spreadsheet and tabulated separately with the same or similar uses grouped together based on whether they were actualised (previously implemented) or proposed (hypothesised uses). Following this, the subsequent step will be to identify what can be termed ‘specific programme theories’, which relate to the explicit and implicit assumptions regarding the method of action for a certain PROMs use. These will include references to existing theories and to also what are termed ‘lay theories’ originating from the specific programmes themselves. Initial rough logic models (IRLMs) were produced outlining the decision-making sequences of the PROMs uses and offered a framework for the coding, identification and organisation of initial programme-based assumptions, about how the identified PROMs uses are purported to function from the perspectives of the included papers.

The final step will involve tracing shared programme-based assumptions between the specific programme theories, allowed for the abstraction, and grouping of specific programme theories to create overarching programme theories. The resulting overarching theories are compound theories of the assumptions, from both a purely theoretical and an actualised perspective, of how PROMs are supposed to function within routine post-stroke clinical practice and patient care. These overarching programme theories once refined form a small number of IRPTs for the purpose of phases two and three of this realist synthesis.

#### Candidate programme theories

The assumptions about how PROMs influence post-stroke care, and clinical practice have been identified under the following four IRPTs:

1. PROMs aid in identifying patient-perceived impact of stroke on health-related domains and quality of life. [*This is especially the case where impacts are not directly observable, e.g. mood, fatigue, or social functioning*]
2. PROMs facilitate shared decision-making (SDM) post-stroke.
3. PROMs allow for the evaluation of the efficacy or impact of treatment.
4. PROMs promote the long-term monitoring of changes over time.

Many of the IRPTs identified operate at and across different phases of the post-stroke pathway in response to micro and meso contexts such as stroke severity or care pathways. Therefore, this conceptualisation is a generalisation for the purpose of offering a simplified conceptualisation. The temporal element of PROMs use will be further explored and elaborated via the refinement of the logic models as part of phase two of this realist synthesis. Further, the IRPTs outlined here are of a provisional nature and will be subject to addition and prioritisation before refinement and testing against primary evidence during the theory-testing phase of this realist synthesis.

#### Stakeholder engagement and IRPT prioritisation

The initial set of IRPTs identified will be subject to stakeholder prioritisation. The stakeholder group will consist of the research team, healthcare professionals with a background in stroke, representatives of stroke-focused third-sector organisations and stroke survivors. Semi-structured facilitated discussions will be utilised as the format of the stakeholder groups via either face-to-face, remote conferencing or a combination of both. Stroke survivors will be contacted through an established patient and public involvement (PPI) forum, and health professionals will be approached through existing professional networks. The professionals will be purposively sampled to be representative of the professionals engaged in stroke care, e.g. doctors, nurses and allied health professionals (occupational therapists, physiotherapists, speech and language therapists, and psychologists). In addition, attempts will be made to recruit a stakeholder group that is representative of the constituent nations of the UK. The stakeholder group will allow for the participants to share knowledge and experiences of the lived experience of stroke and of PROMs for the purpose of theory refinement. The final aspect of the stakeholder group’s contribution during the first stage of this realist synthesis will be the prioritisation of what they consider to be the two most important IRPTs for testing during the second stage of the realist synthesis.

### Phase 2: theory testing

The previously identified IRPTs will serve to direct the testing and refinement of programme theories during phase two of this realist synthesis. Whilst PROMs are the intervention of interest in this review, it is not necessarily PROMs themselves, which will be subject to testing and refinement. Rather, scrutiny falls on the programme theories, which are the ideas or assumptions that underpin PROMs use in post-stroke clinical practice and patient care. Therefore, the second phase of this realist synthesis will search for and utilise the evidence from primary research studies for the purpose of testing and refining the prioritised programme theories relating to how PROMs influence post-stroke clinical practice and patient care.

#### Literature search: theory testing

The multicomponent theory-testing searches will retain components of the theory-building searches, and an indicative set of search concepts is outlined in Table 2. In line with the theory-building literature search, concept A ‘Stroke’ will remain in its original form. Further, concept B ‘PROMs’ will be utilised unchanged, but in conjunction with a novel concept D ‘IRPT’ specifically developed from the IRPTs prioritised by the stakeholder group. Additionally, searches will be run without concept B ‘PROMs’ but with concept D ‘IRPT’, for the purpose of not limiting the realist synthesis solely to results related directly to PROMs and is more fitting to the realist synthesis approach to IRPT investigation. This is of utility, where insufficient explanation can be derived from a specific focus on PROMs alone. The decision to continue with iterative searches of primary studies of non-PROMs interventions with shared programme theories will be taken by the research team, based on the sufficiency of explanatory power within the existing body of evidence related directly to PROMs use. The previously specified concept C ‘Evidence Type’ will not be utilised in the theory-testing searches. In recognition of the heterogeneity of study types and the numerous synonymous terms, the identification and screening to include only primary studies will be achieved through inclusion and exclusion criteria alone as opposed to through search syntax or filters. Nevertheless, any secondary sources of evidence, as previously defined, which are identified by searches will be utilised to locate further primary sources via scrutiny of references.

However, due to the multidisciplinary nature of stroke care and clinical practice, consideration has been paid to include data sources associated with allied health professional research. The search for evidence will take place across the following databases: MEDLINE, EMBASE, PsycINFO (OVID) CINAHL (Ebsco) and Cochrane. The Cochrane systematic review database will be utilised to select and retrieve primary studies. The search syntax will be developed for MEDLINE using a combination of text words and subject headings and will be translated to meet the requirements of each of the databases searched. However, where subject headings are not replicated in different databases, then, synonyms or near neighbour concepts will be utilised as approximations to the meaning of the original syntax. In addition, both forward and backward citation tracking via Google Scholar will be utilised. In contrast to the initial searches for theory building, the theory-testing searches will not include searches of the grey literature, as these are secondary sources.

The process of searching for primary research for theory testing and refinement will end at the point of data saturation or ‘theoretical saturation’ [18], understood as the point in which new evidence does not contribute novel or significant contributions to theory refinement. The point of saturation will be decided by a joint decision of the research team in consideration of the available evidence and the status of the theory under development.

#### Inclusion and exclusion criteria: theory-testing literature search

This realist synthesis will use a two-step process, of abstract followed by full text review against the inclusion criteria to discern article inclusion. During the process of abstract review, where abstracts do not accurately describe the content of a paper, articles will undergo full-text screening. Further, to ensure rigour, a random sample of 20% of all abstracts screened will undergo double screening by two members of the research team independently to establish agreement rates. In the case of discrepancies in the inclusion of the papers between reviewers, inclusion will be decided by discussion and a joint decision of the research team.

The inclusion and exclusion criteria to be utilised in this phase will be set out following the prioritisation of two IRPTs. Nevertheless, the indicative inclusion and exclusion criteria, for this part of the review, outlined in Table 3 will retain much of the focus of the theory-building searches. The key differences that can be outlined a priori in the criteria will be the inclusion of primary as opposed to secondary forms of evidence and that the criteria will be open to sources not directly related to PROMs. In line with the previous section, only sources related directly to PROMs will be included in the initial searches, with the potential for widening of the inclusion criteria based on the IRPTs. The decision to widen the inclusion will be taken by the research team according to the need for greater explanatory power. In similarity to the theory-building phase, the criteria will maintain a focus on sources concentrating on stroke and routine individual clinical practice and patient care. In relation to the exclusion criteria, it is possible to specify at this stage that the theory-testing criteria will exclude studies not involving stroke survivors, studies that focus solely on the development of PROMs or other patient-reported tools and their associated properties (e.g. reliability and validity), and secondary sources such as reviews will also be excluded.

**Table 3:** Inclusion/exclusion criteria—phase 2 theory-testing searches. Note: where concept B ‘PROMs’ is utilised in the theory-testing searches, all studies not relating to PROMs will be excluded as per the previous searches outlined in Table 2

Inclusion	Exclusion
Study has a population clinically diagnosed with any or a mixture of the following conditions: cerebral infarction, non-traumatic intracerebral haemorrhage.	Study population solely focuses on any of the following conditions: transient ischaemic attack (TIA), traumatic brain injury, hypoxic brain injury, subarachnoid haemorrhage.
Study population consists of adult stroke survivors at any period post-clinical diagnosis.	Study consists of a paediatric stroke population.
Studies focused on routine clinical practice or care at the micro- and/or meso-levels. (incl. pragmatic studies)	Studies concentrating on the development of PROs or tools related to the IRPT.
IRPT at the level of the individual, e.g. shared decision-making between a clinician and patient.	Studies concentrating on secondary testing or comparative testing of PROs or tools related to the IRPT.
Interpretation and or feedback of the PROM or tools related to the IRPT necessary.	Studies concentrating on the psychometric properties of PROs or tools related to the IRPT (e.g. validity and reliability).
All study designs.	Studies involving the aggregation of individual scores from PROs or tools related to the IRPT.
English language or high-quality translation available.	Studies in which the scores of PROMs or tools related to the IRPT are an outcome of a research study, including the evaluation of an intervention or observational research exploring trends in domains such as quality of life.
	Any secondary research studies including literature reviews, comments, editorials, etc. (*this type of study design is reserved for phase 1: theory building. However, secondary sources will be followed for the purpose of discovering further primary sources.)*
	Date of publication pre 01/01/00 due to the significant changes to stroke care and services.

#### Data extraction and quality appraisal

##### Data extraction

Articles found to meet the inclusion criteria, will be taken forward for data extraction. Within realist synthesis, the size or proportion of the contribution to theory development will vary from article to article. These contributions, often termed fragments, are extracted from the full text paper due to their relevance to theory testing. The full-text papers will be annotated and coded based on the contributions of identified fragments to the different programme theories being tested. As individual articles may produce many fragments, which can contribute to numerous programme theories, a robust system of coding and cataloguing will be utilised to ensure a systematic approach to data extraction. All extracted data, in the form of fragments, will be stored in an Excel spreadsheet developed specifically for this review, which will catalogue each fragment against the paper it originates and theory it contributes to. The coding process may highlight novel or unexplored IRPTs which will not be further explored during this review. Within realist synthesis, it is possible that articles are subject to further iterative annotation and coding during the testing of programme theories. This being the case, version control will be utilised to ensure that all stages of annotation and coding are captured for the purposes of rigour and transparency.

In tandem with annotation and coding, this review will use a data extraction form, designed specifically for this review, to allow fragments to be accurately catalogued against the programme theories to which they contribute. The first part of the data extraction form will capture the presence or otherwise of key aspects in the reporting of the study such as the research question or questions and descriptions of the intervention, study methodology and setting. This information is necessary to ensure study relevance through the completeness or otherwise of the description of the key elements of the study, which can enhance or detract from the available richness and depth of explanation. The second part of the extraction form relates to context, mechanism, outcome (CMO) extraction. The design of the form allows for the position of the CMO within an article to be noted, as fragments should not be extracted and complied devoid of their original context. Furthermore, the data-extraction tool will detail the specific CMO configuration identified during extraction to ensure that this explanatory relationship is preserved.

Papers will be graded using a scoring system developed specifically for the review. The data-extraction form will grade fragments, based on the richness of their contribution to explanation and causal insight as ‘Good’, ‘Sufficient’ and ‘Insufficient’. The categories relate to the completeness and clarity of the key aspects of the study, whilst also evaluating the CMOs and the richness of causal insight contained in CMO configurations. To ensure consistency and rigour in the grading process, a random sample of 20% of the graded articles will also be independently graded by a second member of the review team to establish agreement rates. In cases of disagreement between reviewers, all disagreements of relevance will be discussed and decided upon by the review team. However, as previously stated, within realist synthesis, the grading of fragments for significance to theory testing does not necessarily reflect the quality or methodological rigour of the fragment from which it originates.

##### Quality appraisal

This review will make use of the Mixed Methods Appraisal Tool (MMAT) [19, 20] to assess the methodological rigour of fragments deemed ‘good’ or ‘sufficient’. This approach to quality appraisal will be of specific use to enhance rigour when the review is testing and refining theory against claims directly made by the fragments. Nevertheless, much of the exploration in realist synthesis is related to unintended outcomes or indirect relationships; therefore, the use of MMAT for quality appraisal will be of lesser importance. In agreement with Pawson et al.’s assertion that ‘the worth of a primary study is determined in the synthesis’ [21], fragments of studies with low methodological rigour are not necessarily excluded from synthesis where they contribute to theory testing and refinement, but this will be considered within the synthesis process.

### Phase 3: synthesis

The ultimate aim of realist synthesis is to create transferable middle-range theory-based explanations for stakeholders to understand how programme theories work for whom, how and under what circumstances [21]. Therefore, the final part of the realist synthesis is the drawing together of fragments from primary research studies in a process of synthesis. The aim of this synthesis is to explain how PROMs influence individual-level post-stroke care and clinical practice through the process of retroduction [22].

The process of synthesis involves the bringing together of the fragments with their competing claims and programme theories. In the following adjudication, the process of synthesis is aiming for an inference to the best explanation (IBE) [15, 23]. The resulting theory derived from IBE would be judged as good where it has the greatest available explanatory power stemming from its ability to satisfy, what are termed, the ‘explanatory virtues’ [24]. These explanatory virtues are (1) coherence (the congruence or coherence between sources) [25], (2) simplicity (the more parsimonious or simpler an explanation, hypothesis or theory is) and (3) unification (the ability to explain more observations or phenomena) [24]. Thus, this form of abductive reasoning seeks the simplest and most likely explanation for the largest amount or number of observations or phenomena and is key tenet of a realist approach. To arrive at theories which best satisfy the IBE criteria, the review team, who will be familiar with the fragments and the programme theories, will judge the competing theory or theories through an iterative process of discussion. Each iteration will be documented through working papers for transparency. The final step in the process of synthesis will involve consulting the stakeholder group, who will offer their perspectives on the final middle range theories and the review team’s IBE justification for selecting these middle range theories in light of the available evidence. This will serve to enhance rigour but also to understand how these theories will be interpreted and taken forward during subsequent knowledge translation.

## Discussion

This realist synthesis will offer theory-based explanations as to how PROMs influence post-stroke clinical practice and patient care. This realist synthesis, like others, presents challenges in two key areas, iterative searching of sources and data extraction. The need to clearly document all searches has been laid out in previous sections; however, it is worth stressing this novel feature of the realist synthesis methodology and the challenges it presents in relation to existing review frameworks. Furthermore, the breadth and depth of data extraction requires systematic handling of fragments with robust systems to ensure fragments are in a utilisable format that will underlie the rigour and robustness of the resulting synthesis. Protocol amendments will be agreed by the review team, documented clearly in a supplement of the protocol, with any important amendments resulting in an update to the review registration via PROSPERO, which maintains an accessible update history.

Considered dissemination will be critical not only via traditional means such as a peer-reviewed journal article and conferences, but also at stakeholder events, which allow for the context-dependent engagement required for meaningful understanding and the avoidance of misinterpretation of what can often be theoretically demanding explanations. Thus, an aspiration of the process of stakeholder involvement in theory prioritisation is to stimulate a wider dialogue in relation to the actualised and hypothesised uses of PROMs post-stroke. The opening of a dialogue with stakeholders regarding the priority areas for PROMs use and development has the potential to concentrate efforts at refining a smaller number of uses than are currently proposed in the literature. Building on this, as Pawson et al. [21] suggest, the need to engage with stakeholders throughout the realist synthesis process is a methodological requirement for a successful application of the methodology. This being the case, continued synthesis is a process of dialogue or linkage [26] that reflexively links the findings of the realist synthesis to the contexts of stakeholders. However, the resulting realist synthesis cannot offer an empirical account to underpin guarantees of success or failure of different PROMs uses, which would be contrary to the ontology of realist methodologies. Thus, realist synthesis, as Pawson et al. [21] describe, offers caveats and considerations such as ‘‘remember A’, ‘beware of B’, ‘take care of C’.’ [21] to aid further implementation and programme refinement within new and existing contexts.

The resulting review will be of practical use for the wider clinical and research community to enact refinements and shifts in the emphasis of PROMs programmes within post-stroke patient care and clinical practice based on the findings of this review. It will also provide a touchstone for further testing and theoretical refinement in subsequent research.

## Supplementary Information

**Additional file 1.** PRISMA-P 2015 Checklist.

**Additional file 2.** Sources of grey literature.

## Data Availability

Data sharing is not applicable to this study as no datasets were generated or analysed during this current study.

## Abbreviations

AuSCR: Australian Stroke Clinical Registry
CINAHL: Cumulative Index to Nursing and Allied Health Literature
CMO: Context, mechanism and outcome
Embase: Excerpta Medica database
IBE: Inference to the best explanation
IRLM: Initial rough logic model
IRPT: Initial rough programme theory
KPI: Key performance indicator
MMAT: Mixed Methods Appraisal Tool
PPI: Patient and public involvement
PRISMA-P: Preferred Reporting Items for Systematic Review and Meta-Analysis Protocols
PRO: Patient-reported outcome
PROM: Patient-reported outcome measure
RAMESES: Realist And Meta-narrative Evidence Syntheses: Evolving Standards
SDM: Shared decision-making
