# Supplementary material for: Patient-reported outcome measures (PROMs) use in post-stroke patient care and clinical practice: a realist synthesis protocol
Source: Syst Rev. 2021 Apr 28;10:128. doi: 10.1186/s13643-021-01682-w (PMC8082773; doi:10.1186/s13643-021-01682-w)
Supplement: Supplementary file 2 — Additional file 2. Sources of grey literature. [file 13643_2021_1682_MOESM2_ESM.docx]

# Additional File 2.

## Sources of grey literature

### Web Domains Searched via Google Advanced Search

https://www.rcplondon.ac.uk/ (Royal College of Physicians)

https://www.rcn.org.uk/ (Royal College of Nurses)

https://www.rcslt.org/ (Royal College of Speech and Language Therapists)

https://www.csp.org.uk/ (Chartered Society Physiotherapy)

https://www.rcot.co.uk/ (Royal College of Occupational Therapists)

https://www.acpin.net/ (Association of Chartered Physiotherapists in Neurology)

https://www.theabn.org/ (Association of British Neurologists)

https://www.basp.org/ (British Association of Stroke Physicians)

https://www.bda.uk.com/ (British Dietetic Association)

https://www.orthoptics.org.uk/ (British and Irish Orthoptic Society)

https://www.bps.org.uk/ (British Psychological Society)

https://www.collegeofparamedics.co.uk/ (College of Paramedics)

https://www.sign.ac.uk/ (Scottish Intercollegiate Guidelines Network)

https://www.nice.org.uk/ (National Institute for Health and Care Excellence)
